# Physical Activity and Health-Related Quality of Life in Kidney Transplant Recipients: A Cross-Sectional Exploratory Study of Clinical and Inflammatory Parameters

**DOI:** 10.3390/healthcare14040545

**Published:** 2026-02-22

**Authors:** Francesca Tinti, Marco Alfonso Perrone, Giulia Bartoli, Maria Josè Ceravolo, Gabriele D’Urso, Roberta Angelico, Luca Salomone, Silvia Lai, Kohei Ashikaga, Paolo Menè, Pasquale Farsetti, Antonino De Lorenzo, Giuseppe Tisone, Ferdinando Iellamo, Anna Paola Mitterhofer

**Affiliations:** 1 Department of Clinical and Molecular Medicine, Sapienza University of Rome, 00189 Rome, Italy; francesca.tinti@uniroma1.it (F.T.);; 2 Nephrology and Dialysis Unit, St. Andrea University Hospital, 00189 Rome, Italy; 3 Department of Clinical Sciences and Translational Medicine, University of Rome Tor Vergata, 00133 Rome, Italy; farsetti@med.uniroma2.it (P.F.); iellamo@uniroma2.it (F.I.); 4 Nephrology and Dialysis Unit, University Hospital Tor Vergata, 00133 Rome, Italymariajose.ceravolo@ptvonline.it (M.J.C.); annapaola.mitter@uniroma2.it (A.P.M.); 5 Department of Surgical Sciences, University of Rome Tor Vergata, 00133 Rome, Italytisone@med.uniroma2.it (G.T.); 6 Nephrology Unit, Department of Translational and Precision Medicine, Umberto I University Hospital Sapienza University of Rome, 00185 Rome, Italy; silvia.lai@uniroma1.it; 7 Department of Sports Medicine, St. Marianna University School of Medicine, Kawasaki 216-8511, Japan; k2ashikaga@marianna-u.ac.jp; 8 Department of Biomedicine and Prevention, University of Rome Tor Vergata, 00133 Rome, Italy; delorenzo@uniroma2.it; 9 Department of Systems Medicine, University of Rome Tor Vergata, 00133 Rome, Italy

**Keywords:** kidney transplantation, physical activity, IPAQ, inflammatory markers, quality of life

## Abstract

**Background/Objectives:** Physical activity (PA) is a modifiable determinant of health and quality of life (QoL) in kidney transplant recipients (KTRs). However, associations between PA, health-related QoL (HRQoL), inflammation, and clinical factors in KTRs remain incompletely defined. The aim was to evaluate PA levels in KTRs and explore their associations with HRQoL, clinical characteristics, and biochemical and inflammatory markers. **Methods:** We conducted a cross-sectional study of 32 stable KTRs (56% male; mean age 54.5 ± 14.2 years). PA was assessed using the International Physical Activity Questionnaire and classified as low (<700 MET-min/week) or high (≥700 MET-min/week) according to IPAQ categorical scoring. HRQoL was evaluated using the SF-36. Associations with demographic, clinical, biochemical (including potassium), and inflammatory markers—including neutrophil-to-lymphocyte ratio, platelet-to-lymphocyte ratio, systemic immune-inflammation index, and ferritin—were analyzed using multivariable binary logistic regression models. **Results:** Sixty-three percent of participants achieved high PA, which was associated with better physical functioning (78.8 vs. 58.3; *p* = 0.016), fewer emotional role limitations, younger age at transplantation, and preemptive transplantation or peritoneal dialysis. Active patients had modestly higher potassium levels (4.61 vs. 4.25 mmol/L; *p* = 0.041), a hypothesis-generating finding that should be interpreted cautiously. Inflammatory indices showed no significant associations. **Conclusions:** Although most KTRs achieved adequate PA levels, physical inactivity persisted in over one-third. Targeted strategies addressing HRQoL and clinical factors may support PA engagement after transplantation.

## 1. Introduction

Chronic kidney disease (CKD) remains a major global health challenge, with end-stage renal disease (ESRD) patients facing high morbidity and mortality despite advances in renal replacement therapies.

Kidney transplantation is the preferred treatment for ESRD, offering superior survival, improved quality of life, and better cost-effectiveness compared to maintenance dialysis [1]. Although immunosuppressive therapy has significantly reduced acute rejection rates and improved short-term graft survival, long-term outcomes remain limited, with transplant recipients continuing to face elevated risks of cardiovascular disease (CVD), metabolic complications, and impaired physical function—factors that significantly impact long-term prognosis [2,3].

Physical activity, as defined by the International Physical Activity Questionnaire (IPAQ), encompasses any skeletal-muscle movement that increases energy expenditure, including occupational, transportation, household, and leisure-time activities [4]. The IPAQ is a reliable and widely used instrument for quantifying physical activity in both research and clinical practice [5]. In CKD, physical activity levels are strongly associated with cardiovascular health, functional capacity, and health-related quality of life (HRQoL) [6]. Following kidney transplantation, maintaining or increasing physical activity becomes even more critical for optimizing long-term health outcomes [7].

The SF-36 Health Survey is a widely validated and psychometrically robust self-report tool designed to assess HRQoL across diverse patient populations [8]. It has been extensively employed in CKD and transplant cohorts to evaluate both physical and mental health dimensions [9].

Although kidney transplantation improves exercise tolerance and overall physiological status, physical activity levels among recipients remain highly variable. Persistent comorbidities, side effects of immunosuppressive medications, chronic low-grade inflammation, fatigue, and safety concerns are among the key factors limiting post-transplant activity [10]. Chronic low-grade inflammation persists in many kidney transplant recipients and contributes to cardiovascular risk, graft dysfunction, and impaired physical performance [11]. Composite hematological indices such as the neutrophil-to-lymphocyte ratio (NLR), platelet-to-lymphocyte ratio (PLR), neutrophil-to-platelet ratio (NPR), and systemic immune-inflammation index (SII) integrate innate immune activation and adaptive immune suppression and have emerged as accessible markers of chronic low-grade inflammation and immune dysregulation in kidney transplant recipients and other chronic inflammatory conditions [12]. These indices have been associated with cardiovascular risk, graft outcomes, and metabolic complications after transplantation, making them suitable candidates for exploring the relationship between physical activity and inflammatory status in this population [13].

Although physical activity has a recognized role in improving cardiovascular and functional outcomes after kidney transplantation, its clinical correlates and relationship with health-related quality of life and inflammatory markers remain underexplored. Recent large-scale studies have investigated physical activity patterns in kidney transplant recipients: a Brazilian cohort of 1105 patients reported that nearly 69% were physically inactive [14], while a German multicenter study involving 676 recipients evaluated the effects of structured exercise interventions on physical fitness outcomes [15]. However, these studies primarily focused on prevalence and interventional programs, rather than on the association between physical activity and individual clinical, biochemical, and inflammatory parameters.

Therefore, we aimed to evaluate physical activity levels in kidney transplant recipients and to explore their associations with health-related quality of life and potentially modifiable clinical and inflammatory factors. We hypothesized that higher levels of physical activity would be associated with better health-related quality of life and potentially more favourable inflammatory profiles, acknowledging the exploratory nature of this analysis.

## 2. Materials and Methods

### 2.1. Study Design

This was a cross-sectional, observational study designed to explore the relationship between physical activity level, indices of quality of life, and clinical and biochemical parameters in kidney transplant recipients. Ethical approval for this study was granted by the Ethics Committee of the University Hospital Tor Vergata (protocol code 60.24) on 21 October 2024. The study was carried out according to the Declaration of Helsinki, and written informed consent was obtained from all participants.

### 2.2. Participants

A total of 32 adult kidney transplant recipients attending the Nephrology Outpatient Clinic of Nephrology and Dialysis Unit and Transplant Centre of University Hospital Tor Vergata, between January and June 2025, were enrolled. Inclusion criteria were:

- Age ≥18 years
- Clinically stable renal graft for at least 6 months
- Absence of severe cardiopulmonary comorbidities

Participants were consecutively recruited from the outpatient kidney transplant clinic of the University Hospital of Tor Vergata (Rome) over a six-month period. Eligible patients were approached during routine follow-up visits. Reasons for non-participation included refusal, lack of time, or incomplete questionnaire completion.

All patients provided informed consent prior to participation in the study.

### 2.3. Variables and Analysis

Demographic, clinical, and biochemical data were retrieved from each participant’s medical records and included for analysis.

Blood samples were collected during post-transplant follow-up visits at the Transplant Center, in fasting conditions, prior to pharmacological therapy administration, and processed according to traditional standards.

All biochemical and haematological analyses were performed in the certified central laboratory of Tor Vergata University Hospital using standardized automated methods routinely adopted in clinical practice. Complete blood counts were obtained using automated haematology analysers, and derived inflammatory indices (neutrophil-to-lymphocyte ratio, platelet-to-lymphocyte ratio, neutrophil-to-platelet ratio, and systemic immune-inflammation index) were calculated from absolute cell counts. These markers were selected to reflect chronic, low-grade inflammation rather than acute-phase inflammatory responses.

Ferritin and standard biochemical parameters were measured using validated immunochemical and enzymatic assays in accordance with internal quality control procedures and laboratory accreditation standards.

On the same day, a medical check-up was performed during which physical activity indices and quality of life were assessed. This approach ensures that the study variables reflect a contemporaneous snapshot of the patients’ status in a standardized clinical setting.

### 2.4. Physical Activity and Health-Related Quality of Life Assessment

Health-related quality of life was assessed using the 36-Item Short Form Survey (SF-36).

Physical activity was assessed using the validated Italian short form of the International Physical Activity Questionnaire (IPAQ-SF) [16], which evaluates the frequency and duration of vigorous, moderate-intensity, and walking activities performed for at least 10 consecutive minutes during the past 7 days. Total physical activity was calculated as MET-minutes/week according to official IPAQ scoring guidelines (vigorous activity × 8.0 METs + moderate activity × 4.0 METs + walking × 3.3 METs) and categorized using instrument-specific thresholds: inactive (<700 MET-min/week), sufficiently active (700-2519 MET-min/week), and active/very active (>2520 MET-min/week) [17].

The SF-36 evaluates eight domains of health, including physical functioning, bodily pain, general health, vitality, social functioning, role limitations due to physical and emotional problems, and mental health. Perceived physical functioning (HRQoL outcome) was evaluated using the Physical Functioning subscale of the validated Italian SF-36 Health Survey, which measures patients’ self-reported capacity to perform daily physical activities (e.g., walking, climbing stairs, self-care). The results of the SF-36 were reported separately for each of the eight domains. Scores were calculated according to standard scoring procedures.

Patients self-completed the two questionnaires during routine follow-up visits. The two instruments served complementary purposes: IPAQ-SF quantified actual physical activity behavior, while SF-36 Physical Functioning assessed functional capacity and its perceived impact on quality of life.

### 2.5. Activity Level Categorization

Participants were categorized into two groups based on total weekly MET-minutes:

- High activity: ≥700 MET-min/week
- Low activity: <700 MET-min/week

Although IPAQ guidelines propose three categories, we adopted a pragmatic clinical dichotomization to distinguish physically inactive/low-active recipients from those achieving adequate activity levels, aligning with our focus on inactivity as a modifiable cardiovascular risk factor rather than dose–response modelling.

In line with the original IPAQ categorical scoring and with previous IPAQ-based studies in Italian and nephrology/transplant cohorts, we classified participants with ≥700 MET-min/week as having higher physical activity, while those with <700 MET-min/week were classified as having lower activity [17]. This cut-off corresponds to an IPAQ-derived category threshold and is conceptually consistent with the WHO minimum recommendation of ≥600 MET-min/week for adults [18].

Physical activity was assessed using the validated Italian IPAQ-SF.

### 2.6. Statistical Analysis

Given the substantial dispersion and presence of potential outliers in some measured variables, we assessed the distribution of these variables for normality using the Shapiro–Wilk test. Data transformations (logarithmic and square root) were applied where appropriate to improve normality; however, for variables that remained non-normally distributed, non-parametric statistical methods were employed to ensure robust analysis.

Between-group comparisons were performed using independent-samples *t*-tests or Mann–Whitney U tests, as appropriate.

The relationships between continuous variables were examined using Pearson’s or Spearman’s correlation coefficients, depending on the normality of the data distribution.

We performed binary logistic regression analyses to investigate the association between high physical activity levels, defined as IPAQ ≥ 700 MET-min/week, versus lower activity levels, and selected demographic, anthropometric, functional, and inflammatory parameters.

Because the outcome variable was dichotomized into two groups based on physical activity level (IPAQ ≥ 700 vs. <700), binary logistic regression was used to calculate odds ratios (OR). This approach was appropriate for the binary nature of the dependent variable. Multinomial logistic regression (MLR) was not applied, as it is typically reserved for dependent variables with more than two categories.

Separate models were tested, each including gender, age at transplantation (years), BMI (kg/m^2^), and physical functioning score, together with one inflammatory marker (systemic immune-inflammation index [SII], platelet-to-lymphocyte ratio [PLR], neutrophil-to-platelet ratio [NPR], or neutrophil-to-lymphocyte ratio [NLR]).

Model fit was evaluated using deviance, Akaike Information Criterion (AIC), and McFadden’s pseudo-R^2^. Odds ratios (OR) with 95% confidence intervals (CI) and *p*-values were reported for all predictors.

This study was exploratory in nature, with the sample size determined by feasibility and the availability of eligible patients. Given the absence of prior data on expected effect sizes and variance for our specific population, a formal power calculation was not possible. We acknowledge this limitation; the results should be interpreted as hypothesis-generating, and will inform the design of future confirmatory and adequately powered studies.

All statistical analyses were conducted using Jamovi version 2.6 (Computer Software), retrieved from https://www.jamovi.org, based on R Core Team (2024) [19]. A two-tailed *p*-value < 0.05 was considered statistically significant.

## 3. Results

### 3.1. Population Characteristics

A total of 32 kidney transplant recipients were included in the study, comprising 18 males (56%) and 14 females (44%).

Of the 32 participants, 62.5% (20/32) had IPAQ ≥ 700 MET-min/week (95% CI 45–79%). This proportion was not significantly different from 50% (binomial test, *p* = 0.14), though it trends toward being a majority. Conversely, about 37.5% remain below recommended levels of physical activity.

There were no significant differences in physical activity (IPAQ scores) between male and female participants (2011.5 vs. 1815.1 MET-min/week, respectively). No significant between-group differences were found for body composition and metabolic parameters (BMI, obesity, lipids, hemoglobin) according to physical activity level.

When patients were stratified by physical activity threshold (IPAQ < 700 vs. ≥700 MET-min/week), those with higher activity levels tended to be younger (median age: 56.0 years vs. 52.5 years) and had a younger age at transplantation (41.5 vs. 52.6 years), although this difference did not reach statistical significance (*p* = 0.096 and *p* = 0.075, respectively).

Physical activity levels were influenced by the dialysis modality before transplantation. Patients who had peritoneal dialysis (PD) or preemptive transplantation were more likely to have higher IPAQ values compared to those who underwent hemodialysis (HD) (*p* = 0.041). Conversely, no significant association was found between donor type (cadaveric vs. living) and physical activity levels.

At the time of assessment, all participants were receiving standard maintenance immunosuppressive therapy. Patients were receiving low-dose maintenance corticosteroids, with a median daily prednisone-equivalent dose of 5 mg/day and limited variability across participants (interquartile range 5–7.5 mg/day). Although steroid dosage was not specifically analyzed in relation to physical activity due to limited variability, chronic corticosteroid exposure may influence muscle function and exercise tolerance and should be considered when interpreting physical activity levels in kidney transplant recipients

The data for comparison are summarized in Table 1.

#### 3.1.1. Physical Activity and SF-36 Domains

Physical activity levels were significantly associated with certain SF-36 quality-of-life domains. Physical functioning scores were significantly higher in participants with IPAQ ≥ 700 compared to those with IPAQ < 700 (78.8 vs. 58.3; *p* = 0.016) (Supplementary Figure S1).

Other SF-36 domains, including role limitations (physical and emotional), vitality, social functioning, pain, and general health, did not differ significantly between the two groups (Table 2).

**Figure 1:**
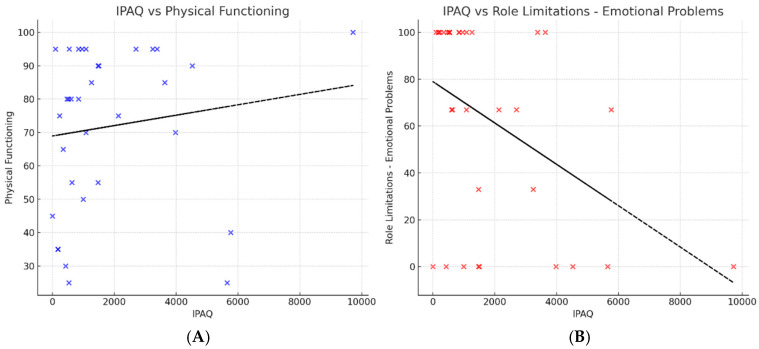
Association between physical activity and SF-36 domains. Scatter plots display the relationship between IPAQ total MET-min/week and SF-36 subscales, with fitted regression lines illustrating the direction of association. Panel (**A**) shows a positive association between IPAQ scores and Physical Functioning (Spearman ρ = 0.370, *p* = 0.037), indicating that more physically active kidney transplant recipients report better perceived physical functioning. Panel (**B**) shows a negative association between IPAQ scores and Role Limitations due to Emotional Problems (Spearman ρ = −0.435, *p* = 0.013), suggesting that higher physical activity is related to fewer emotional constraints in daily activities.

Spearman correlation analysis further explored the relationships between physical activity and SF-36 domains. Physical activity (IPAQ) was positively correlated with Physical Functioning (Rho = 0.370, *p* = 0.037) and negatively correlated with Role Limitations due to Emotional Problems (Rho = −0.435, *p* = 0.013), indicating that more physically active recipients reported fewer emotional constraints in daily activities. No significant correlations were observed with the other SF-36 domains (Figure 1).

Figure 1 illustrates the data points with notable dispersion and potential outliers at both extremes. Despite attempts at data transformation, some variables retained skewed distributions, justifying the use of non-parametric tests for comparisons. This approach ensures reliability of the findings despite heterogeneity in the data.

#### 3.1.2. Clinical and Biochemical Variables

There were no significant associations between IPAQ scores and renal function (eGFR or creatinine) or hemoglobin levels. Stratification by hemoglobin threshold (≥10.5 g/dL vs. <10.5 g/dL) also revealed no significant differences in physical activity.

Among biochemical parameters, potassium levels were slightly higher in the IPAQ ≥ 700 group (4.61 [4.4–4.8] vs. 4.25 [3.9–4.5] mmol/L; *p* = 0.041) (Figure S2).

No significant differences were found in iron, PTH, vitamin D, triglycerides, and glucose among groups.

#### 3.1.3. Inflammatory Indices

A weak negative trend was observed between IPAQ and ferritin (Spearman’s ρ = −0.283, *p* = 0.162) and TyG index (ρ = −0.176, *p* = 0.335), but these did not reach statistical significance.

The analysis of inflammatory markers (NLR, PLR, NPR, and SII) showed no statistically significant differences between the low and high physical activity groups:

- NLR (3.46 in low activity vs. 2.37 in high activity, *p* = 0.135)
- PLR (0.174 in low activity vs. 0.127 in high activity, *p* = 0.279)
- NPR (22.4 in low activity vs. 18.1 in high activity, *p* = 0.250)
- SII (150,355 in low activity vs. 140,847 in high activity, *p* = 0.614)

However, the lower activity group (<700) tended to have higher NLR and NPR values, indicating a possible link between lower physical activity and a pro-inflammatory state

**Figure 2:**
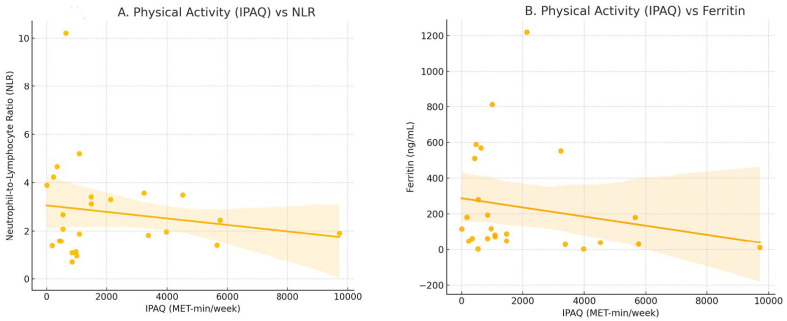
Associations between physical activity (IPAQ MET-min/week) and inflammatory markers in kidney transplant recipients. Scatter plots show the relationship between total physical activity (IPAQ MET-min/week) and (**A**) neutrophil-to-lymphocyte ratio (NLR) and (**B**) ferritin levels, with fitted regression lines indicating the direction of association. Although no statistically significant correlations were detected, lower IPAQ values were generally associated with higher NLR and ferritin, suggesting a trend toward a more pro-inflammatory profile in less active patients.

Figure 2 illustrates the associations between physical activity (IPAQ MET-min/week) and inflammatory markers in kidney transplant recipients.

#### 3.1.4. Multivariable Analysis

A total of four binary logistic regression models were tested, each including sex, age at transplantation, BMI, physical functioning score, and one inflammatory marker (SII, PLR, NPR, or NLR). All models were specified a priori using the same set of covariates (sex, age at transplantation, BMI, and SF-36 Physical Functioning), selected based on clinical relevance, and to avoid overfitting given the limited sample size. Each inflammatory index (NLR, PLR, NPR, SII) was entered separately to minimize collinearity due to shared cellular components. Among them, the model including the neutrophil-to-lymphocyte ratio (NLR) showed the best fit (deviance = 19.2, AIC = 31.2, McFadden’s pseudo-R^2^ = 0.512) (Supplementary Table S1).

Important factors such as comorbidities, time from transplantation and immunosuppressive regimen were considered potential confounders. These variables were evaluated for inclusion in the statistical models; however, based on preliminary analyses and data availability, only predictors demonstrating significant associations were retained to ensure model parsimony.

In this model, higher age at transplantation was associated with a lower likelihood of high physical activity levels (OR = 0.83, 95% CI 0.68–1.03, *p* = 0.091), suggesting that each year younger at transplant was associated with a 16% increase in the likelihood of higher physical activity. Physical functioning showed a positive but non-significant association (OR = 1.07, 95% CI 0.99–1.16, *p* = 0.088). NLR was inversely associated with high physical activity, although not reaching statistical significance (OR = 0.21, 95% CI 0.03–1.45, *p* = 0.113), indicating a trend toward greater physical activity in patients with lower systemic inflammation (Table 3).

**Figure 3:**
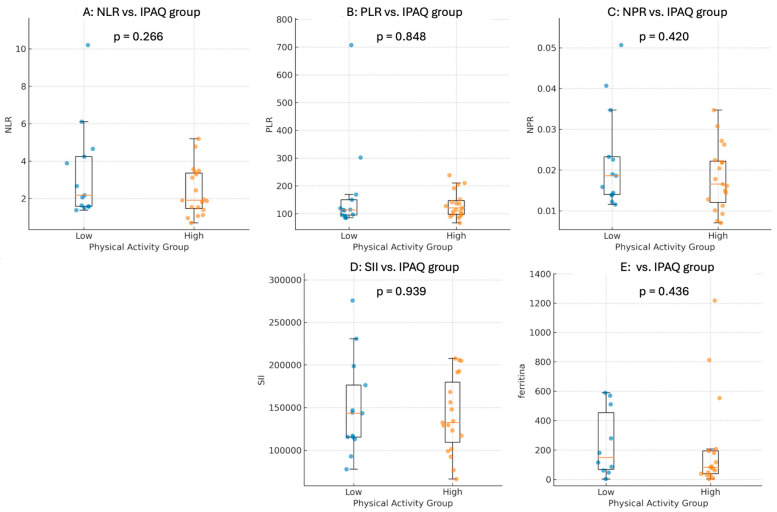
Inflammatory indices and ferritin by physical activity group in kidney transplant recipients. Boxplots show median and IQR; whiskers indicate 1.5 × IQR. Dots represent individual patients. *p*-values are from Mann–Whitney U tests comparing Low vs. High activity: (**A**) NLR, (**B**) PLR, (**C**) NPR, (**D**) Systemic Immune-Inflammation Index (SII = neutrophils × platelets/lymphocytes), and (**E**) Ferritin.

Across all tested models—including SII, PLR, NPR, NLR, and ferritin—all inflammatory markers showed a consistent trend in the same direction, although none reached statistical significance; effect sizes were close to the null with wide confidence intervals, suggesting no meaningful predictive value in this sample. As visually apparent in Figure 3, the distributions of inflammatory indices and ferritin in low and high activity groups substantially overlapped, reinforcing the absence of clinically meaningful separation between categories in this small cohort.

Gender and BMI were not significantly associated with the likelihood of engaging in higher levels of physical activity (*p* = 0.100 and 0.754, respectively). In the final model, being male was associated with a higher odds ratio, but with wide confidence intervals and a *p*-value above the conventional threshold for significance, indicating substantial uncertainty in the estimate. Similarly, BMI showed no meaningful relationship with physical activity, with an odds ratio close to 1 and non-significant results.

## 4. Discussion

In this study, we evaluated physical activity (IPAQ) and health-related quality of life (HRQoL, assessed by SF-36) in kidney transplant recipients and investigated their associations with clinical and biochemical parameters, including systemic inflammatory markers.

### 4.1. Physical Activity Levels in Our Cohort

In our cohort, 62.5% of kidney transplant recipients achieved adequate physical activity levels. This finding is consistent with previous reports in transplant populations and suggests a relatively favorable activity profile in stable recipients.

From a clinical perspective, identifying the substantial proportion of patients who remain inactive may help target personalized interventions aimed at improving long-term outcomes [17].

This finding is clinically relevant, as regular physical activity has been consistently linked to reduced cardiovascular risk, enhanced graft function, and improved quality of life in kidney transplant populations [20,21,22].

Regular physical activity is recognized as a modifiable determinant of cardiovascular risk, graft survival, and health-related quality of life in kidney transplant recipients [21,22,23]. Nonetheless, over one-third of our population, 37.5%, remains below recommended levels and did not reach recommended thresholds, which could potentially increase their risk for cardiovascular events, sarcopenia and reduced functional outcomes.

The study’s design features IPAQ dichotomization at 700 MET-min/week—a validated clinical threshold in transplant populations that optimizes discrimination between high-risk inactive patients and those achieving guideline-concordant activity levels. In kidney transplant populations, physical inactivity itself—rather than incremental increases in activity intensity—has been more consistently associated with adverse cardiovascular outcomes and mortality. Evidence supporting a linear or progressive reduction in cardiovascular risk across increasing IPAQ activity categories remains limited and inconsistent in this specific population [23]. Accordingly, our approach aimed to identify patients at risk due to inactivity rather than to assess incremental benefits across activity intensities.

The IPAQ-derived categorization has been widely used in epidemiological and clinical studies, including Italian cohorts and studies in patients with chronic kidney disease and transplant recipients.

We acknowledge that the World Health Organization (WHO) recommends ≥600 MET-min/week as the minimum level of physical activity for adults to achieve health benefits. However, the WHO threshold represents a public health recommendation, whereas the IPAQ cut-offs are part of a validated methodological framework designed to classify individuals into activity categories for research purposes [18]. For methodological consistency and comparability with previous IPAQ-based studies, we therefore used the ≥700 MET-min/week cut-off to define higher physical activity.

Our results show that higher physical activity levels are associated with better physical functioning, as measured by SF-36, but show no significant association with most biochemical or inflammatory indices. These findings underline the need for systematic screening of physical activity levels in transplant follow-up and targeted interventions, such as supervised exercise programs, to enhance long-term health outcomes.

### 4.2. Low Physical Activity Subgroup

In our cohort, 37.5% of patients exhibited low physical activity levels (<700 MET min/week). This subgroup showed a trend toward older age (mean 58.9 vs. 50.0 years), which is consistent with previous findings that age-related sarcopenia and comorbidities can reduce physical function in kidney transplant recipients [20,21,22,23,24]. Our findings suggest a trend toward higher physical activity levels among recipients transplanted at a younger age, with an estimated increase in the odds of achieving higher activity for each year younger at transplantation; however, this association did not reach statistical significance. Notably, a recent study reported that low muscle mass predicts early hospital readmission after transplantation [25,26], while another demonstrated that pre-transplant sarcopenia is associated with both reduced graft survival and impaired physical recovery [27].

### 4.3. Physical Activity and Quality of Life

Consistent with previous studies, we observed that kidney transplant recipients with higher physical activity levels (IPAQ ≥ 700 MET-min/week) exhibited significantly better physical functioning (SF-36) compared to those with lower activity. This result aligns with reports by Squires et al. [28] and van White et al. [29], which demonstrated that physical activity is a key determinant of functional capacity and perceived physical health among transplant populations.

The positive correlation between IPAQ and physical functioning (ρ = 0.37, *p* = 0.037) supports the notion that even moderate increases in activity can have measurable benefits in this population [16]. Interestingly, we also found a negative correlation between physical activity and role limitations due to emotional problems, suggesting that active patients may experience fewer psychosocial barriers. This interplay between physical and mental well-being has been observed in other chronic disease cohorts and may reflect both the physiological and psychological benefits of regular activity [30].

Although the cross-sectional design precludes causal inference, this association may reflect the well-established relationship between physical activity and psychological well-being, including reduced emotional distress, improved self-efficacy, and better coping strategies in individuals with chronic conditions. In kidney transplant recipients, improved emotional functioning may facilitate greater engagement in daily activities and social participation, potentially reinforcing an active lifestyle.

### 4.4. Physical Activity and Biochemical Variables

We found no significant relationships between physical activity and renal function (eGFR) or haemoglobin levels, which aligns with the understanding that physical activity post-transplant is largely determined by behavioural and lifestyle factors rather than kidney function per se.

The only biochemical variable showing a significant difference was serum potassium, which was slightly higher in more active patients (4.61 vs. 4.25 mmol/L), while remaining within the normal range. This finding may reflect differences in muscle mass, dietary intake, or overall functional status rather than a pathological alteration. Previous studies have reported associations between whole-body potassium content and muscle strength, suggesting that potassium levels may serve as an indirect marker of muscle-related parameters [31,32]. However, as muscle mass and ion transport mechanisms were not directly assessed in the present study, this observation should be considered hypothesis-generating. Further studies in larger cohorts, including direct assessment of body composition and muscle function, are needed to clarify the clinical relevance of this finding. Importantly, no causal inference can be drawn, and the clinical significance of this modest difference remains uncertain. Further studies in larger cohorts, incorporating direct measures of body composition and physical performance, are needed to determine whether serum potassium may serve as a meaningful functional or metabolic correlate of physical activity in kidney transplant recipients.

Although previous studies have linked anaemia and nutritional status to reduced activity in CKD and dialysis patients these associations appear attenuated post-transplant, likely due to improved metabolic and haematological parameters following successful transplantation [10,33].

### 4.5. Inflammation and Physical Activity

Patients with lower physical activity levels (<700 MET-min/week) showed a trend toward higher inflammatory markers. Although we did not observe statistically significant associations between IPAQ and inflammatory markers (NLR, PLR, NPR, SII), this pattern may indicate a potential relationship between sedentary behavior and low-grade inflammation. This is consistent with evidence that sedentary behaviour is associated with a pro-inflammatory phenotype in both healthy and clinical populations [34,35,36], suggesting a potential association between sedentary behavior and markers of low-grade inflammation in this cohort. These observations support further investigation of inflammation as a potentially modifiable factor in post-transplant care.

While physical activity is known to exert anti-inflammatory effects in the general population, the absence of clear associations in our cohort may be related to the small sample size or to the relatively low-grade and heterogeneous inflammation seen in stable transplant recipients. Some trends were noted, with higher NLR and NPR values in the less active group, which might indicate a subtle relationship between inflammation and activity that a larger study could confirm.

These findings highlight the importance of early identification of patients at risk of physical inactivity, especially older recipients and those with subclinical inflammation, as this may have long-term implications for cardiovascular health, muscle preservation, and quality of life. Future studies should further explore the potential anti-inflammatory role of physical activity in kidney transplant recipients.

In this cohort, despite a comprehensive inflammatory assessment, no statistically significant associations were observed between physical activity levels and inflammatory markers (NLR, PLR, NPR, SII). However, patients with lower physical activity levels showed a consistent trend toward higher NLR, NPR, and ferritin values. These findings do not support a definitive association between physical activity and systemic inflammation but may reflect the limited sample size and the low-grade, heterogeneous inflammatory profile of kidney transplant recipients. Rather, these results highlight the need for larger, longitudinal studies to better clarify the relationship between physical activity and low-grade inflammation after kidney transplantation.

### 4.6. Impact of Dialysis History and Transplant Characteristics

The result that patients who underwent peritoneal dialysis or preemptive transplantation are more active than those who had hemodialysis after transplantation may reflect differences in pre-transplant physical conditioning and overall health status.

Our findings are consistent with emerging evidence suggesting potential benefits of preemptive transplantation and peritoneal dialysis on functional and clinical outcomes. Recent studies, including that by Pape et al. [15], have highlighted improved patient-centered outcomes and reduced treatment-related burden associated with these approaches, supporting their possible role in preserving physical functioning. Nevertheless, these observations should be interpreted cautiously and warrant confirmation in prospective studies.

Donor type (cadaveric vs. living) did not influence physical activity levels in our sample, suggesting that post-transplant factors such as lifestyle habits and rehabilitation programs may play a more critical role than donor characteristics.

### 4.7. Clinical Implications

Our findings emphasize the need for targeted interventions to promote physical activity in kidney transplant recipients, particularly in those with low baseline activity. Structured exercise programs have been shown to improve not only physical capacity but also HRQoL and potentially reduce cardiovascular risk—a leading cause of morbidity in this population [37].

Compared to the Brazilian study [14] and the German intervention trial [15], which focused primarily on prevalence and structured exercise programs, our study provides an exploratory analysis linking self-reported physical activity levels with both health-related quality of life (SF-36) and systemic inflammatory indices. Although smaller in size, our cohort allowed a detailed clinical and biochemical characterization, adding insights into potential biological correlates of physical inactivity post-transplantation.

Differences between our findings and those reported in previous studies may be partly explained by variations in patient selection, clinical stability, and exposure to structured rehabilitation or exercise programs. Large cohort studies have often included heterogeneous transplant populations and, in some cases, focused on formal training interventions or long-term fitness programs, whereas our study reflects physical activity behaviour assessed during routine follow-up in clinically stable recipients. Moreover, differences in training dose, assessment tools, and post-transplant care pathways may contribute to discrepancies across studies and should be considered when interpreting comparative results.

Given the observed correlation between activity and physical functioning, clinicians should consider routine screening for physical inactivity (e.g., with IPAQ) and integrate personalized exercise counselling into post-transplant care.

Routine assessment of physical activity using brief, validated instruments such as the International Physical Activity Questionnaire may represent a feasible approach to identify kidney transplant recipients at risk of inadequate physical activity. Previous studies have highlighted the clinical utility of structured physical activity screening in nephrology populations, supporting its potential integration into routine clinical follow-up [22,38,39].

From a clinical perspective, the International Physical Activity Questionnaire (IPAQ) represents a simple and inexpensive tool that can be easily incorporated into routine post-transplant follow-up to identify patients with low physical activity levels.

Patients screening below recommended thresholds may benefit from personalized counseling, referral to supervised or home-based exercise programs, and closer monitoring of functional and metabolic parameters. Such an approach could facilitate early identification of modifiable risk factors and support individualized strategies aimed at improving long-term physical functioning and quality of life in kidney transplant recipients.

### 4.8. Future Research Directions

Several future research directions emerge from this study. Longitudinal studies are needed to clarify the temporal relationship between physical activity, quality of life, and inflammatory status in kidney transplant recipients. Future studies should aim to overcome the limitations of self-reported physical activity by incorporating objective measures such as accelerometers or wearable devices, which may provide more precise estimates of activity patterns and sedentary behavior. 

In addition, more detailed inflammatory profiling, including cytokines and myokines, may help elucidate the biological mechanisms linking physical activity and low-grade inflammation in this population.

Finally, interventional studies testing personalized exercise programs based on individual clinical characteristics may further define physical activity as a modifiable determinant of long-term transplant outcomes

### 4.9. Limitations

This study has several limitations. First, its cross-sectional design limits the ability to infer causal relationships between physical activity and health-related quality of life (HRQoL) or inflammatory status. Second, the relatively small sample size may have reduced the statistical power to detect associations with biochemical and inflammatory variables. Moreover, the single-center design may limit generalizability to transplant populations in different healthcare systems or regions, where demographics, immunosuppression protocols, or activity patterns may vary.

Therefore, results should be interpreted as exploratory and hypothesis-generating.

Finally, physical activity was assessed using the International Physical Activity Questionnaire (IPAQ), a validated tool but one that relies on self-reporting and is subject to recall and social desirability biases, particularly in sedentary individuals and clinical populations such as kidney transplant recipients. Misclassification of physical activity levels may have attenuated true associations with biochemical or inflammatory markers and may partly explain the absence of statistically significant findings. Therefore, some observed associations—particularly those involving HRQoL domains—could reflect reporting bias rather than a direct causal relationship.

Future studies incorporating objective measures of physical activity, such as accelerometry, are warranted to more accurately characterize activity patterns and their biological correlates in this population.

## 5. Conclusions

In this cross-sectional exploratory study, higher physical activity levels in kidney transplant recipients were associated with better physical functioning and selected clinical characteristics, including younger age at transplantation. Modestly higher potassium levels within the normal range were observed in more active patients and may reflect underlying differences in muscle mass or metabolic regulation rather than pathological processes. Although no significant associations with inflammatory indices were detected, less active patients showed consistent trends toward higher inflammatory markers, suggesting a potential relationship between physical inactivity and low-grade inflammation. Notably, a substantial proportion of recipients did not achieve recommended physical activity levels, underscoring the relevance of routine assessment and individualized interventions.

Routine assessment of physical activity using brief questionnaires such as IPAQ may support personalized post-transplant care.

Further longitudinal studies with objective activity measures are warranted to clarify the role of physical activity as a modifiable factor in post-transplant care.

## Figures and Tables

**Table 1:** Characteristics of the 32 kidney transplant recipients according to physical activity level.

Variable	Low Activity IPAQ < 700 (*n* = 12)	High Activity IPAQ ≥ 700 (*n* = 20)	Cohen’s d or OR	95% CI	*p*-Value
Demographics					
Gender Male	5 (15.6)	13 (40.6)	2.60	0.60–11.3	0.277
Current age (years)	56.0 (50.5–70.0)	52.5 (34.8–61.0)	0.63	−0.11–1.36	0.096 **
Age at transplant (years)	53 (48–61)	46 (25–56)	0.68	−0.06–1.41	0.072 **
Time from transplant (years)	3.0 (1.0–9.0)	5.0 (2.8–13.0)	−0.25	−0.96–0.48	0.298 *
BMI (kg/m^2^)	25.0 (23.7–26.9)	24.0 (22.7–25.9)	0.02	−0.72–0.75	0.321 *
Clinical characteristics					
Nephropathy			n.a	n.a	0.349
Glomerulonephritis	2 (6.3)	6 (18.8)			
Congenital disease	3 (9.4)	8 (25.0)			
Other	4 (12.5)	2 (6.3)			
Unknown	3 (9.4)	4 (12.5)			
Hypertension	10 (32.3)	16 (51.6)	0.94	0.13–6.63	0.948
Diabetes mellitus	2 (6.9)	5 (17.2)	2.08	0.33–13.1	0.665
Dyslipidaemia	6 (18.8)	9 (28.1)			0.690
Obesity	2 (6.3)	2 (6.3)	1.94	0.18–21.1	0.581
Type of transplant (Living/Deceased Donor)	4 (12.5)/8 (25.0)	8 (25.0)/12 (37.5)	1.33	0.30–5.96	1.000
Use of Tacrolimus	10 (31.3)	16 (50.0)	0.80	0.12–5.20	1.000
Use of Everolimus	7 (22.6)	5 (16.1)	0.26	0.06–1.19	0.130
Use of steroids	11 (36.7)	12 (40.0)	0.18	0.02–1.76	0.193
RRT before Transplant			n.a.	n.a.	**0.038**
Pre-emptive tx	0	3			
Hemodialysis	12	12			
Peritoneal dialysis	0	5			
Biochemical Parameters					
Serum Creatinine (mg/dL)	1.50 (1.04–1.98)	1.48 (1.16–2.09)	−0.27	−0.99–0.45	0.668 *
eGFR CKD-EPI 2021 (mL/min/1.73 m^2^)	40.0 (32.0–77.0)	51.0 (33.8–70.3)	−0.06	−0.79–0.68	0.951 *
ACR (mg/g)	68 (25–99)	18 (0–379)	−0.39	−1.17–0.39	0.643 **
Haemoglobin (g/dL)	12.6 (10.7–13.6)	12.3 (11.4–13.8)	−0.143	−0.86–0.57	0.697 **
Tacrolimus level	5.0 (4.1–6.6)	6.1 (4.9–7.3)	−0.46	−1.26–0.35	0.391 *
Potassium (mEq/L)	4.3 (3.9–4.5)	4.6 (4.4–4.8)	−0.90	−1.66–−0.15	0.041 *
Calcium (mg/dL)	10.1 (9.5–10.3)	10.0 (9.8–10.5)	−0.17	−0.88–0.55	0.654 **
Magnesium (mg/dL)	1.7 (1.4–2.0)	1.7 (1.6–1.9)	−0.26	−0.98–0.47	0.685 *
PTH (pg/mL)	165 (110–278)	126 (78–150)	0.01	−0.70–0.73	0.167 *
Vitamin D (ng/mL)	16 (13–24)	25 (16–31)	−0.57	−1.31–0.17	0.131 **
Serum iron (µg/dL)	69 (42–99)	56 (32–839)	0.16	−0.57–0.90	0.457 *
Ferritin (ng/mL)	182 (62–511)	83 (39–195)	0.14	−0.67–0.94	0.419 *
Inflammatory indexes					
TyG	4.69 (4.57–4.86)	4.66 (4.50–4.90)	−0.01	−0.72–0.70	0.974 **
NLR	2.37 (1.58–4.34)	1.90 (1.46–3.34)	0.57	−0.18–1.30	0.269 *
PLR	0.11 (0.09–0.15)	0.12 (0.09–0.15)	0.41	−0.33–1.13	1.000 *
NPR	17.2 (14.0–26.1)	16.6 (12.1–22.2)	0.43	−0.30–1.16	0.535 *
SII	130,278 (114,897–179,817)	132,337 (109,435–179,817)	0.19	−0.54–0.91	0.614 **

Data are expressed as median (interquartile range) for continuous variables and *n* (%) for categorical variables. High physical activity was defined as an International Physical Activity Questionnaire (IPAQ) score ≥ 700 MET-min/week and low physical activity as IPAQ < 700 MET-min/week. Abbreviations: n.a., not available; BMI, body mass index; eGFR, estimated glomerular filtration rate; ACR, albumin-to-creatinine ratio; PTH, parathyroid hormone; TyG, triglyceride–glucose index; NLR, neutrophil-to-lymphocyte ratio; PLR, platelet-to-lymphocyte ratio; NPR, neutrophil-to-platelet ratio; SII, systemic immune-inflammation index; RRT, renal replacement therapy. * Mann–Whitney. ** T-Student. Bold for statistically significant value.

**Table 2:** SF-36 Domains and Clinical/Biochemical Characteristics/variables by IPAQ of 32 patients stratified by high and low physical activity level.

SF-36 Domain	Low Activity IPAQ < 700 (*n* = 12)	High Activity IPAQ ≥ 700 (*n* = 20)	Cohen’s d	95% CI	*p*-Value
Physical Functioning	60.0 (35.0–80.0)	87.5 (70.0–95.5)	−0.92	−1.67–0.16	0.016 *
Role Limitations—Physical	87.5 (25.0–100)	50.0 (25.0–100)	0.12	−0.60–0.87	0.715 *
Role Limitations—Emotional	100.0 (67.0–100)	67.0 (0.0–100)	0.62	−0.12–1.35	0.091 *
Energy/Fatigue	70.0 (53.8–81.3)	67.5 (53.8–76.3)	0.04	−0.68–0.75	0.800 *
Emotional Well-being	72.0 (62.0–86.0)	66.0 (59.0–77.0)	−0.05	−0.77–0.67	0.754 *
Social Functioning	63.0 (43.8–91.0)	63.0 (50.0–75.0)	0.07	−0.65–0.78	0.708 *
Pain	100 (45.0–100)	71.5 (58.0–88.0)	0.08	−0.64–0.80	0.486 *
General Health	55.0 (38.8–68.5)	50.0 (37.5–55.0)	0.28	−0.45–0.99	0.492 *

Data are presented as median (interquartile range). High physical activity was defined as an International Physical Activity Questionnaire (IPAQ) score ≥ 700 MET-min/week and low physical activity as IPAQ < 700 MET-min/week. * Mann–Whitney.

**Table 3:** Logistic regression model with neutrophil-to-lymphocyte ratio (NLR) as an inflammatory predictor of high physical activity (IPAQ ≥ 700 MET-min/week).

Predictor	OR	95% CI for OR	*p*-Value
Male gender	581.04	0.2987–1.13 × 10^6^	0.100
Age at transplant (years)	0.834	0.6756–1.028	0.091
Physical functioning score	1.072	0.9898–1.160	0.088
BMI (kg/m^2^)	0.961	0.7485–1.230	0.754
Neutrophil-to-lymphocyte ratio (NLR)	0.212	0.03108–1.450	0.113

Binary logistic regression assessing demographic, anthropometric, functional, and inflammatory predictors of high physical activity (coded as 1) compared with lower activity (coded as 0). Odds Ratios (OR) > 1 indicate higher odds of high activity; OR < 1 indicate lower odds. CI = confidence interval. IPAQ = International Physical Activity Questionnaire; BMI = Body Mass Index; NLR = neutrophil-to-lymphocyte ratio.

## Data Availability

No new data were created or analyzed in this study.

## Supplementary Materials

The following supporting information can be downloaded at: https://www.mdpi.com/article/10.3390/healthcare14040545/s1, Figure S1: Scatter plot illustrating the relationship between IPAQ scores and SF-36 Physical functioning among kidney transplant recipients; Figure S2. Scatter plot illustrating the relationship between IPAQ scores and potassium levels among kidney transplant recipients; Table S1: Model Fit Statistics for Logistic Regression Models.

## Abbreviations

The following abbreviations are used in this manuscript:

CKD	Chronic kidney disease
ESRD	End-stage renal disease
CVD	Cardiovascular disease
IPAQ	International Physical Activity Questionnaire
HRQoL	health-related quality of life
NLR	neutrophil-to-lymphocyte ratio
PLR	platelet-to-lymphocyte ratio
NPR	neutrophil-to-platelet ratio
SF-36	36-Item Short Form Survey
MET	metabolic equivalent
SII	immune-inflammation index
PD	Peritoneal dialysis
HD	Hemodialysis

## References

[1] Leonardis F., Gitto L., Favi E., Oliva A., Angelico R., Mitterhofer A.P., Cacciola I., Santoro D., Manzia T.M., Tisone G. (2023). A Keynesian perspective on the health economics of kidney transplantation would strengthen the value of the whole organ donation and transplantation service. Front. Public Health.

[2] Zelle D.M., Corpeleijn E., Stolk R.P., de Greef M.H., Gans R.O., van der Heide J.J.H., Navis G., Bakker S.J. (2011). Low physical activity and risk of cardiovascular and all-cause mortality in renal transplant recipients. Clin. J. Am. Soc. Nephrol..

[3] Reggiani F., Moroni G., Ponticelli C. (2022). Cardiovascular risk after kidney transplantation: Causes and current approaches to a relevant burden. J. Pers. Med..

[4] Craig C.L., Marshall A.L., Sjöström M., Bauman A.E., Booth M.L., Ainsworth B.E., Pratt M., Ekelund U., Yngve A., Sallis J. (2003). International Physical Activity Questionnaire: 12-country reliability and validity. Med. Sci. Sports Exerc..

[5] Sember V., Meh K., Sorić M., Starc G., Rocha P., Jurak G. (2020). Validity and reliability of International Physical Activity Questionnaire (IPAQ) in office-working adults: A cross-sectional study. Int. J. Environ. Res. Public Health.

[6] Bozkurt H.N., Yıldırım M., Çavdar C., Bildacı Y.D. (2024). Physical activity parameters as determinants of cardiovascular disease risk in kidney transplant recipients: An accelerometer-based study. Adv. Interv. Cardiol..

[7] Mathur S., Janaudis-Ferreira T., Hemphill J., Cafazzo J.A., Hart D., Holdsworth S., Lovas M., Wickerson L. (2021). User-centered design features for digital health applications to support physical activity behaviors in solid organ transplant recipients: A qualitative study. Clin. Transplant..

[8] Ware J.E., Sherbourne C.D. (1992). The MOS 36 Item Short Form Health Survey (SF 36): I. Conceptual framework and item selection. Med. Care.

[9] Fletcher B.R., Damery S., Aiyegbusi O.L., Anderson N., Calvert M., Cockwell P., Ferguson J., Horton M., Paap M.C.S., Sidey-Gibbons C. (2022). Symptom burden and health-related quality of life in chronic kidney disease: A global systematic review and meta-analysis. PLoS Med..

[10] van Adrichem E.J., Dekker R., Krijnen W.P., Verschuuren E.A.M., Dijkstra P.U., van der Schans C.P. (2018). Physical activity and sedentary time in transplant recipients. Phys. Ther..

[11] Alotaibi M., Trollinger B., Kant S. (2024). Management of kidney transplant recipients for primary care practitioners. BMC Nephrol..

[12] Li X., Wang L., Liu M., Zhou H., Xu H. (2024). Association between neutrophil-to-lymphocyte ratio and diabetic kidney disease in type 2 diabetes mellitus patients: A cross-sectional study. Front. Endocrinol..

[13] Lo C.N., Wong N.E.J.W., Ho S., Ang E.J.H., Leung B.P. (2025). Evaluating the effects of exercise on inflammation markers in musculoskeletal pain: A systematic review and meta-analysis. Sports.

[14] Sertorio E.S., Colugnati F.A.B., Denhaerynck K., De Smet S., Medina J.O.P., Reboredo M.M., De Geest S., Sanders-Pinheiro H. (2024). ADHERE BRAZIL Study Team. Factors Associated With Physical Inactivity of Recipients of a Kidney Transplant: Results From the ADHERE BRAZIL Multicenter Study. Phys. Ther..

[15] Pape L., Boeck H.T., Boyen J., Nöhre M., Schiffer L., Kück M., Schieffer E., Albrecht A., de Zwaan M., Tegtbur U. (2025). Physical activity and exercise performance in patients after kidney transplantation—Data from the prospective KTx360° aftercare program. J. Sci. Med. Sport.

[16] Mannocci A., Di Thiene D., Del Cimmuto A., Masala D., Boccia A., De Vito E. (2010). International Physical Activity Questionnaire: Validation and assessment in an Italian sample. Ital. J. Public Health.

[17] Hreńczuk M., Wasińska E., Małkowski P. (2024). Physical activity levels in transplant recipients. Ann. Transplant..

[18] World Health Organization (2020). WHO Guidelines on Physical Activity and Sedentary Behaviour.

[19] R Core Team (2024). R: A Language and Environment for Statistical Computing.

[20] Roi G., Stefoni S., Mosconi G., Brugin E., Burra P., Ermolao A., Granito M., Macini P., Mastrosimone S., Nacchia F. (2014). Physical activity in solid organ transplant recipients: Organizational aspects and preliminary results of the Italian project. Transplant. Proc..

[21] Tzvetanov I., West-Thielke P., D’Amico G., Johnsen M., Ladik A., Hachaj G., Grazman M., Heller R., Fernhall B., Daviglus M. (2014). A novel and personalized rehabilitation program for obese kidney transplant recipients. Transplant. Proc..

[22] Calella P., Hernández Sánchez S., Garofalo C., Ruiz J.R., Carrero J.J., Bellizzi V. (2019). Exercise training in kidney transplant recipients: A systematic review. J. Nephrol..

[23] Heiwe S., Jacobson S.H. (2011). Exercise training for adults with chronic kidney disease. Cochrane Database Syst. Rev..

[24] Painter P., Roshanravan B. (2013). The association of physical activity and physical function with clinical outcomes in adults with chronic kidney disease. Curr. Opin. Nephrol. Hypertens..

[25] Wang W. (2024). Optimizing quality of life in kidney transplant recipients through structured exercise: A systematic review and evidence-based guidelines. Med. Sci. Monit..

[26] Wong L., Kent A.B., Lee D., Roberts M.A., McMahon L.P. (2022). Low muscle mass and early hospital readmission post kidney transplantation. Int. Urol. Nephrol..

[27] Karakizlis H., Trudel N., Brose A., Reinisch A., Reichert M., Hecker A., Bender F., Askevold I., Rainer L., Weimer R. (2023). Sarcopenia of kidney transplant recipients as a predictive marker for reduced graft function and graft survival after kidney transplantation. Langenbeck’s Arch. Surg..

[28] Squires R.W., Bonikowske A.R. (2022). Cardiac rehabilitation for heart transplant patients: Considerations for exercise training. Prog. Cardiovasc. Dis..

[29] White R.L., Vella S., Biddle S., Sutcliffe J., Guagliano J.M., Uddin R., Burgin A., Apostolopoulos M., Nguyen T., Young C. (2024). Physical activity and mental health: A systematic review and best-evidence synthesis of mediation and moderation studies. Int. J. Behav. Nutr. Phys. Act..

[30] Messina G., Alioto A., Parisi M.C., Mingrino O., Di Corrado D., Crescimanno C., Kuliś S., Sahin F.N., Padua E., Canzone A. (2023). Experimental study on physical exercise in diabetes: Pathophysiology and therapeutic effects. Eur. J. Transl. Myol..

[31] Sucharita S., Sreenath N., Shinjini B., Raj Tony D.S., Rebecca K. (2023). Reference values for muscle mass and strength in healthy Indian adults using whole-body potassium counter and isokinetic dynamometer. Indian J. Public Health.

[32] Vigh-Larsen J.F., Frangos S.M., Overgaard K., Holloway G.P., Mohr M. (2025). Fatiguing high-intensity intermittent exercise depresses maximal Na^+^/K^+^-ATPase activity in human skeletal muscle assessed using a novel NADH-coupled assay. Pflügers Arch.-Eur. J. Physiol..

[33] Czaja-Stolc S., Chatrenet A., Potrykus M., Ruszkowski J., Torreggiani M., Lichodziejewska-Niemierko M., Dębska-Ślizień A., Piccoli G.B., Małgorzewicz S. (2024). Adipokines and myokines as markers of malnutrition and sarcopenia in patients receiving kidney replacement therapy: An observational, cross-sectional study. Nutrients.

[34] Pinto A.J., Bergouignan A., Dempsey P.C., Roschel H., Owen N., Gualano B., Dunstan D.W. (2023). Physiology of sedentary behavior. Physiol. Rev..

[35] Roshanravan B., Robinson-Cohen C., Patel K.V., Ayers E., Littman A.J., de Boer I.H., Ikizler T.A., Himmelfarb J., Katzel L.I., Kestenbaum B. (2013). Association between physical performance and all-cause mortality in CKD. J. Am. Soc. Nephrol..

[36] Wang M., Pan W., Xu Y., Zhang J., Wan J., Jiang H. (2022). Microglia-mediated neuroinflammation: A potential target for the treatment of cardiovascular diseases. J. Inflamm. Res..

[37] Billany R.E., Bishop N.C., Castle E.M., Graham-Brown M.P.M., Greenwood S.A., Lightfoot C.J., Wilkinson T.J. (2025). Physical activity interventions in adult kidney transplant recipients: An updated systematic review and meta-analysis of randomized controlled trials. Ren. Fail..

[38] Polara G., Montagnoli A., Palazzo R., Orlandi M., Mascherini G., Corsi M., Falconi E., Stefani L. (2024). Cardiorespiratory Performance in Kidney and Liver Transplant Recipients: The Dilemma to Combine Lifestyle and Fitness. J. Funct. Morphol. Kinesiol..

[39] Ponticelli C., Favi E. (2021). Physical Inactivity: A Modifiable Risk Factor for Morbidity and Mortality in Kidney Transplantation. J. Pers. Med..

