# Supplementary material for: Physical Activity and Health-Related Quality of Life in Kidney Transplant Recipients: A Cross-Sectional Exploratory Study of Clinical and Inflammatory Parameters
Source: Healthcare (Basel). 2026 Feb 22;14(4):545. doi: 10.3390/healthcare14040545 (PMC12940355; doi:10.3390/healthcare14040545)
Supplement: Supplementary file 1 [file healthcare-14-00545-s001.zip › healthcare-4103672-supplementary.pdf]

Figure S1. Scatter plot illustrating the relationship between IPAQ scores and SF-36 Physical functioning among kidney transplant recipients.

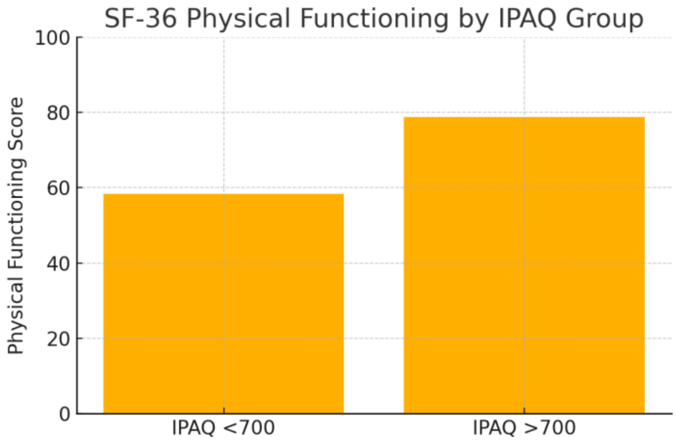

Physical functioning score resulted higher in active patients IPAQ > 700 (p=0.016)

Figure S2. Scatter plot illustrating the relationship between IPAQ scores and potassium levels among kidney transplant recipients.

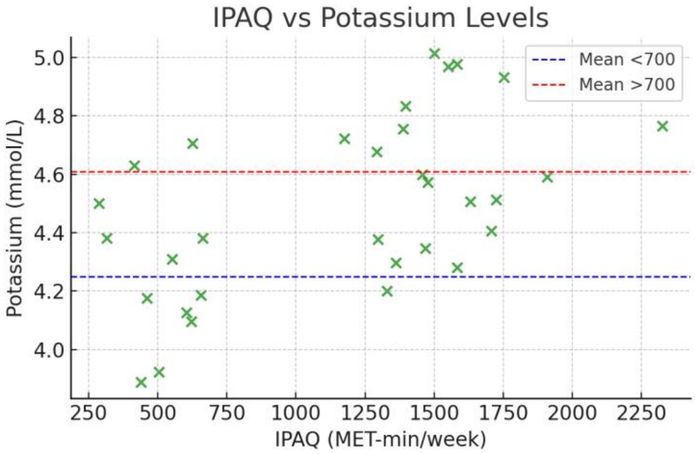

Higher levels of potassium in the IPAQ ≥700 group (4.61 vs. 4.25 mmol/L; p = 0.041)

Table S1. Model Fit Statistics for Logistic Regression Models

Model (Inflammatory Index)	−2 Log Likelihood	AIC	McFadden R^2
NLR	19.2	31.2	0.512
PLR	24.1	36.1	0.389
NPR	22.6	34.6	0.427
SII	29.4	36.4	0.380

Note: Lower AIC and deviance indicate better model fit. All models included sex, age at transplantation, BMI, and SF-36 Physical Functioning as covariates.
